# Serum zonulin levels are increased in Alzheimer’s disease but not in vascular dementia

**DOI:** 10.1007/s40520-023-02463-2

**Published:** 2023-06-19

**Authors:** Elisa Boschetti, Giacomo Caio, Carlo Cervellati, Anna Costanzini, Valentina Rosta, Fabio Caputo, Roberto De Giorgio, Giovanni Zuliani

**Affiliations:** 1 Cellular Signalling Laboratory, Department of Biomedical and Neuro Motor Sciences (DIBINEM), Institute of Human Anatomy, University of Bologna, Via Irnerio, 48, 40126 Bologna, Italy; 2 Department of Translational Medicine, University of Ferrara, Ferrara, Italy

**Keywords:** Aging, Alzheimer’s disease, Blood–brain barrier, Dementia, Gut-brain-microbiota axis, Permeability

## Abstract

**Background:**

Zonulin is involved in the integrity and functioning of both intestinal-epithelial barrier and blood–brain barrier (BBB) by regulating tight junction molecular assembly.

**Aim:**

Since changes in microbiota and BBB may play a role in neurodegenerative disorders, we aimed to determine whether serum zonulin levels change in older patients affected by different types of dementia or mild cognitive impairment (MCI).

**Methods:**

We evaluated serum zonulin levels in patients with late-onset AD (LOAD), vascular dementia (VAD), MIXED (AD + VAD) dementia, amnestic MCI, and in healthy controls.

**Results:**

Compared with controls, serum zonulin increased in LOAD, MIXED dementia, and aMCI but not in VAD, independent of potential confounders (ANCOVA *p* = 0.01; LOAD vs controls, *p* = 0.01; MIXED vs. controls, *p* = 0.003; aMCI vs. controls, *p* = 0.04). Notably, aMCI converting to dementia showed significantly higher levels of zonulin compared with stable aMCI (*p* = 0.04). Serum zonulin inversely correlated with the standardized Mini-Mental State Examination (MMSE) score (*p* < 0.05), regardless of potential confounders.

**Discussion:**

We found increased serum zonulin levels in patients with aMCI, LOAD and MIXED dementia, but not in VAD; moreover, zonulin levels were higher in aMCI converting to AD compared with stable ones.

**Conclusions:**

Our findings suggest that a dysregulation of intestinal-epithelial barrier and/or BBB may be an early specific event in AD-related neurodegeneration.

## Introduction

Zonulin is a 47 KDa protein corresponding to pre-haptoglobin identified by Fasano et al. [1, 2]. It is considered the human counterpart of the *zonula occludens* toxin, an enterotoxin synthesized by Vibrio cholera that impairs the intestinal epithelial barrier via the reversible opening of the tight junctions (TJs) [3]. In the gut, TJs are multiprotein complexes that seal the intercellular space between adjacent enterocytes. TJs are “dynamic doors” regulated by physiological/pathological stimuli (e.g. inflammation, TJ components imbalance) causing assembly or disassembly (sealing or opening) of the intercellular space [4]. So far, zonulin is the only human protein known to reversibly regulate intestinal permeability by modulating intercellular TJs [5]. The cellular release of zonulin transactivates EGF-receptor through proteinase-activated receptor 2 (PAR-2), leading to protein kinase C-alpha (PKC-α) dependent tight junction disassembly. This effect is associated with a transient displacement of *zonula occludens* 1, along with occludin dissociation, actin polymerization and myosin-1C phosphorylation [2, 6, 7].

Although the intestinal-epithelial barrier (IEB) and the blood-brain barrier (BBB) are composed of different cell types, (epithelial and endothelial, respectively) the paracellular permeability of both is regulated by TJs that are sensitive to the disruption induced by inflammatory mediators [8–11]. As previously hypothesized [12], the disruption of TJs at the BBB and IEB may be triggered by a mutual mechanism underlying central nervous system (CNS) disorders that have been associated with the gut-brain axis. Previous works demonstrated that in vitro zonulin enhances the permeability of BBB and IEB; moreover, it has been shown that zonulin up-regulation occurs in brain tumors, and that this feature correlates with the degree of malignancy and BBB impairment [13]. By interfering with endothelial cell assembly, zonulin can also induce transmigration of neuronal progenitors across the BBB in glioma cell line [14]. Taken together, this evidence suggests that zonulin-dependent mechanisms, by impairment of the BBB, might contribute to the pathogenesis of neurodegenerative disorders, including Alzheimer’s disease (AD) [15]. In support of this hypothesis, recent data in AD patients provide a possible link associating the intestine to the CNS via a pathway referred to as the “gut-brain-microbiota axis” [16, 17]. Intestinal dysbiosis can result in a zonulin-dependent increased permeability of the gut barrier leading to neuroinflammation and neurodegeneration [16]. The ageing process and related low-grade inflammation have been also associated with the “leaky gut”, which is a potential source of inflammation and dysbiosis related to geriatric syndromes [18]. Despite the growing interest in this topic, epidemiological and clinical data on the association between zonulin, AD, and other types of dementia are still rare.

In the present study, we evaluated serum zonulin levels in elder patients with late-onset AD (LOAD), vascular dementia (VAD), mixed dementia (LOAD + VAD − MIXED) and compared them to healthy controls. Moreover, in order to check whether changes in zonulin levels might precede dementia onset, we evaluated its levels also in subjects with amnestic MCI (aMCI) which are at high risk of developing AD [19].

## Materials and methods

### Patients and samples

A total of 159 subjects (65-89 years), referred to the Center for Cognitive Decline and Dementia (CCDD) of the University Internal Medicine, Arcispedale S. Anna, Ferrara, Italy, were consecutively enrolled in the study. The herein reported retrospective study sample included:

- 40 patients (70–89 years) with mild/moderate probable LOAD diagnosed by the NIA-AA workgroups criteria [20]. Standardized Mini-Mental State Examination (MMSE) from Molloy and Standish [21] range: 18–23; Clinical Dementia Rating (CDR) range: 1–2;
- 18 patients (67–88 years) with probable VAD, according to the NINDS-AIREN criteria [22]. MMSE range: 16–24; CDR range: 1–2;
- 42 patients (71–85 years) with MIXED dementia: in these patients, a definite diagnosis of LOAD or VAD was not possible since they showed both clinical/instrumental characteristics of VAD (e.g., significant vascular disease and/or focal neurological signs) and AD (memory impairment, type of progression, brain atrophy). MMSE range: 16–24; CDR range: 1–2;
- 36 patients (68–87 years) with aMCI defined as a documented deficit in memory, without (aMCI single domain) or with (aMCI multiple domains) impairment in other cognitive domains, in a patient who did not meet the clinical criteria for dementia [23]. MMSE range: 25–29.

**Fig. 1:**
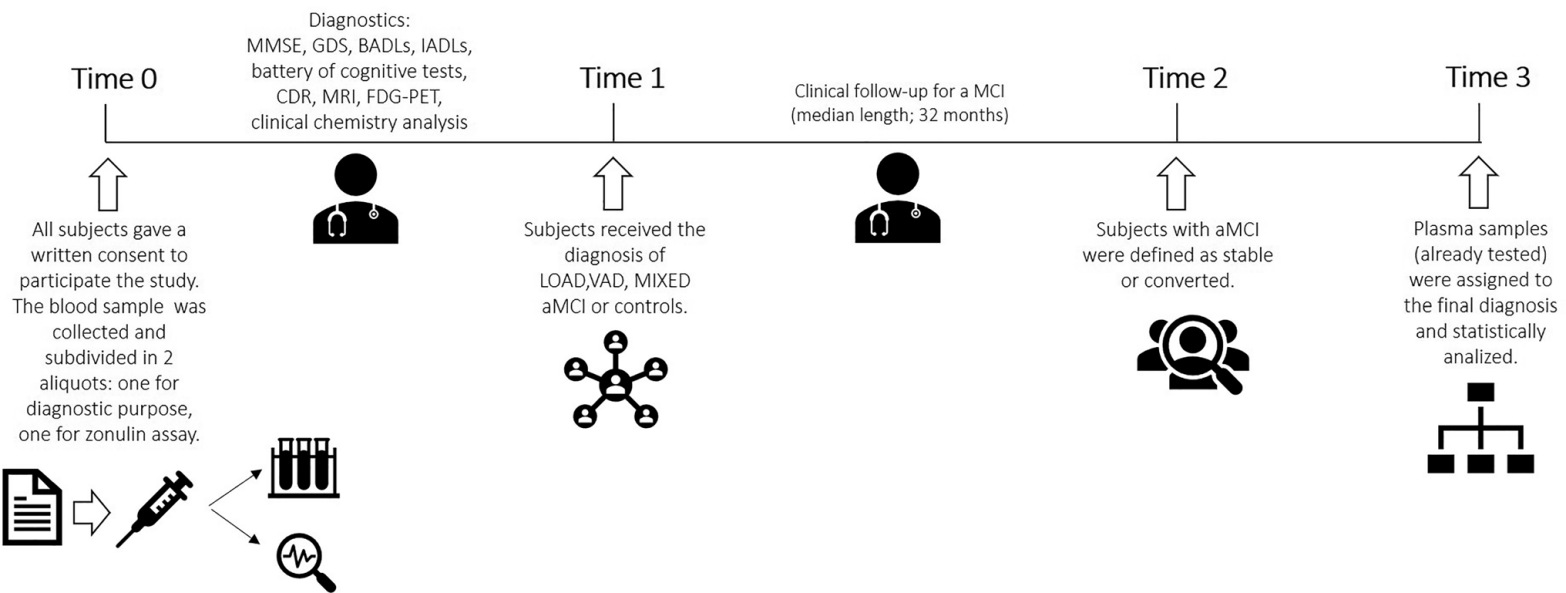
Experimental timeline. Subjects were seen once before diagnosis (first visit; time = 0). At that time, for those who agreed to participate to the study, two blood samples were obtained: one for routine diagnostic tests; the other for zonulin concentration assay. The collected samples were all analysed in a blinded condition, with no connection to the patients’ diagnosis. Patients and controls were subsequently divided into the different groups (diagnosis; time 1). The aMCI group was the only one to undergo a follow-up evaluation, which was of variable length depending on the time of the enrolment. Over a 32-month median follow-up, we identified two subsets of aMCI patients: those who progressed to overt dementia; or those who remain stable (Sub-diagnosis aMCI; time 2). Finally, the levels of zonulin were assigned to the corresponding patients/group and analysed (Data analysis; time 3)

Twenty-three subjects (65-87 years) with any evidence of dementia and functional disabilities attributable to cognitive impairment, served as controls (CTR). MMSE range: 27-30. CTR were geriatric subjects; thus this group served to normalize zonulin variations described in “Healthy Aging” [24]. Among older (≥ 65 years) patients evaluated between 2018 and 2022 at the CCDD of the Department of Internal Medicine, S. Anna University Hospital, Ferrara (Italy), n= 159 entered the present study. Inclusion criteria were identical to those indicated in a previously published investigation from our group (see [25]). Subjects and controls gave a written informed consent to participate in the study (study protocol number: 170579 - Azienda Ospedaliero-Universitaria S. Anna, Ferrara, Italy) and two blood samples were collected for routine diagnostic purposes and zonulin assay. Once the diagnosis was formulated patients were subdivided in the respective groups, i.e. LOAD, VAD, MIXED or aMCI. Those without any evidence cognitive impairment served as controls. Over a 32-month median follow-up period, n=18 patients progressed to dementia (referred to as “aMCI converted”, while 20 remained stable (“aMCI stable”). A timeline specifying enrolment, diagnosis and sampling is shown in Fig. 1.

At the time of the first visit, when the blood sample was collected, there was no evidence of acute illnesses. None of the subjects was in treatment with non-steroidal anti-inflammatory drugs, antibiotics, or steroids at the time of recruitment. General and neuropsychological examinations, including MMSE, geriatric depression scale, and CDR, were carried out as previously described [26–28]. The functional status was evaluated by basic and instrumental activities of daily living [29, 30]. Demographic and medical history data (e.g., hypertension, coronary heart disease, stroke, diabetes, chronic obstructive pulmonary disease) were collected by trained personnel.

All patients who received a diagnosis of dementia different from those included in the study were excluded. Only patients without signs and symptoms of gastrointestinal diseases were included in the study. Clinical chemistry analyses were routinely performed, including serum B12 vitamin, folate, liver, kidney and thyroid function tests, blood cell count, and arterial oxygen saturation to exclude causes of secondary cognitive impairment.

### Serum zonulin measurement

The peripheral blood samples were collected by venipuncture into Vacutainer^®^ tubes without anticoagulant after overnight fasting. After 30 min of incubation at room temperature, the blood samples were centrifuged at 4650 ×g for 20 min. The sera were collected and stored at −80 ℃ in single-use aliquots until analysis. Total proteins were quantified using a NanoDrop 2000 spectrophotometer (Thermo Scientific, Milan, Italy). Zonulin concentration was assayed using a specific Zonulin ELISA Kit (Cusabio, Hubei, China) following the manufacturer instructions (detection range: 0.625–40 ng/mL; sensitivity: 0.156 ng/mL) using 100 µl of serum/well as starting material. Each sample was analysed in duplicate and zonulin concentration was normalised to total protein and reported as pg of zonulin/mg total protein (zonulin/TP) ± standard error (SE).

### Statistical analysis

Statistical analyses were performed using the statistical package IBM SPSS Statistics software (SPSS) for Windows (version 13.0). All continuous variables were first analyzed for normal distribution using Kolmogorov–Smirnov and Shapiro–Wilk tests. Variables with significant deviation from normal distribution were log-transformed before entering statistical analyses. Normal variables (including those approaching normality upon log-transformation) were expressed as mean ± standard deviation (SD), while non-normally distributed variables were expressed as median (Inter Quartile Range). Categorical variables were expressed as a percentage within the group. Comparison of more than two groups was performed by using ANOVA (Sidak post-hoc) and Kruskal–Wallis (pairwise comparison by Dunn’s Multiple Comparison) for normal and non-normal variables, respectively. The Chi-square test was used for comparing categorical variables. The outliers in these analyses were detected first by converting the data to z-scores, and then by evaluating whether the absolute z-value was higher than three. Associations between continuous normal and non-normal variables were tested by assessing Pearson’s coefficient and Spearman’s correlation, respectively. Multivariate regression analysis (stepwise backward) was used to test the independence of the correlation between log-serum zonulin and variable correlated at univariate analysis (MMSE, years of education, hemoglobin, fasting glucose and FT4); age was forced into the model. Analysis of covariance (ANCOVA) was used to test whether the differences revealed at univariate analysis were independent of potential confounders. Finally, differences in zonulin levels were evaluated using the Kruskal-Wallis test coupled with Dunn’s Multiple Comparison as a post-test and expressed as mean ± SE.

## Results

### Cross-sectional data: serum zonulin levels in controls vs. aMCI, LOAD, MIXED, and VAD

The main characteristics of the samples according to the cognitive diagnosis are shown in Table 1. Patients with LOAD or VAD were significantly older than CTR (*p*<0.05). Female gender was prevalent in both LOAD and VAD vs CTR (*p*<0.05), and in VAD compared to aMCI and MIXED dementia (*p*<0.05). Formal education was significantly lower in all groups of patients compared to CTR (*p*<0.05), whereas smokers were more frequent in LOAD and VAD (*p*<0.05). As regards comorbidities, hypertension was more prevalent among patients with VAD and aMCI (*p*<0.05), although diabetes was more frequent in MIXED dementia and aMCI (*p*<0.05). Coronary artery disease was more common in MIXED dementia (*p*<0.05); stroke/TIA were more prevalent among patients with VAD and MIXED dementia (*p*<0.05).

**Table 1:** Main clinical and laboratory characteristics and serum zonulin levels in patients with different types of dementia, aMCI, and in healthy controls

	CTR (n = 23)	LOAD (n = 40)	VAD (n = 18)	MIXED (n = 42)	aMCI (n = 36)
Characteristics					
Age (years)	76 ± 5	79.5 ± 5^a^	81 ± 5^a^	78 ± 4	78 ± 4
Female gender	45%	60%^a^	71%^a^	52%^c^	51%^c^
Formal education (years)*	11 (6–13)	5 (4–8)^a^	5 (2–6)^a^	5 (5–8)^a^	5 (4–8)^a^
MMSE score (/30)	28 (26–30)	21 (19–23)^a,e^	22 (20–24)^a,e^	19 (17–22)^a,e^	25 (23–27)^a^
Current smoker	4%	12%^a^	10%^a^	6%	5%^b^
Clinical chemistry parameters					
Hemoglobin (g/dL)	13.3 ± 1.6	13.4 ± 1.5	13.2 ± 1	13 ± 1.7	13 ± 1.5
Creatinine (mg/dL)	0.9 ± 0.2	0.8 ± 0.1	1.0 ± 0.4	0.9 ± 0.3	0.9 ± 0.1
Fasting glucose (mg/dL)	96 ± 14	95 ± 14	99 ± 17	98 ± 20	97 ± 22
Total cholesterol (mg/dL)	199 ± 39	198 ± 37	225 ± 45	198 ± 48	193 ± 38
hs-CRP	0.12 (0.04–0.32)	0.26 (0.16–0.50)^a^	0.10 (0.04–1.06)	0.11 (0.05–0.24)	0.12 (0.08–0.38)
FT4	1.0 ± 0.2	1.2 ± 0.2	1.0 ± 0.2	1.1 ± 0.2	1.2 ± 0.2
TSH	2.2 ± 1.3	2.3 ± 1.6	2.0 ± 0.8	2.5 ± 3.3	2.2 ± 1.3
Comorbidities					
Hypertension	47%	54%	94%^a,b^	60%	75%^a^
Diabetes	10%	13%	7%	17%^c^	17%^c^
CHD	14%	13%	8%	17%^c^	15%
Stroke/TIA	3%	0%	14%^a,b,e^	10%^a,b^	0%

Normally distributed variables are expressed as mean ± SD, while non-normally distributed variables are indicated as median (Interquartile Range)

Categorical variables are expressed as a percentage within groups

Statistical analysis: ANOVA for normal variables; Kruskall–Wallis for non-normal variables; Chi-squared for categorical variables

*MMSE* standardized mini-mental state examination, *TSH* thyroid stimulating hormone, *FT4* free thyroxine, *hs-CRP* high-sensitivity C-reactive protein, *CHD* coronary heart disease

Pairwise comparison: ^a^*p* < 0.05 vs. Controls; ^b^*p* < 0.05 vs. LOAD; ^c^*p* < 0.05 vs. VAD; ^d^*p* < 0.05 vs. MIXED; ^e^*p* < 0.05 vs. aMCI

**Fig. 2:**
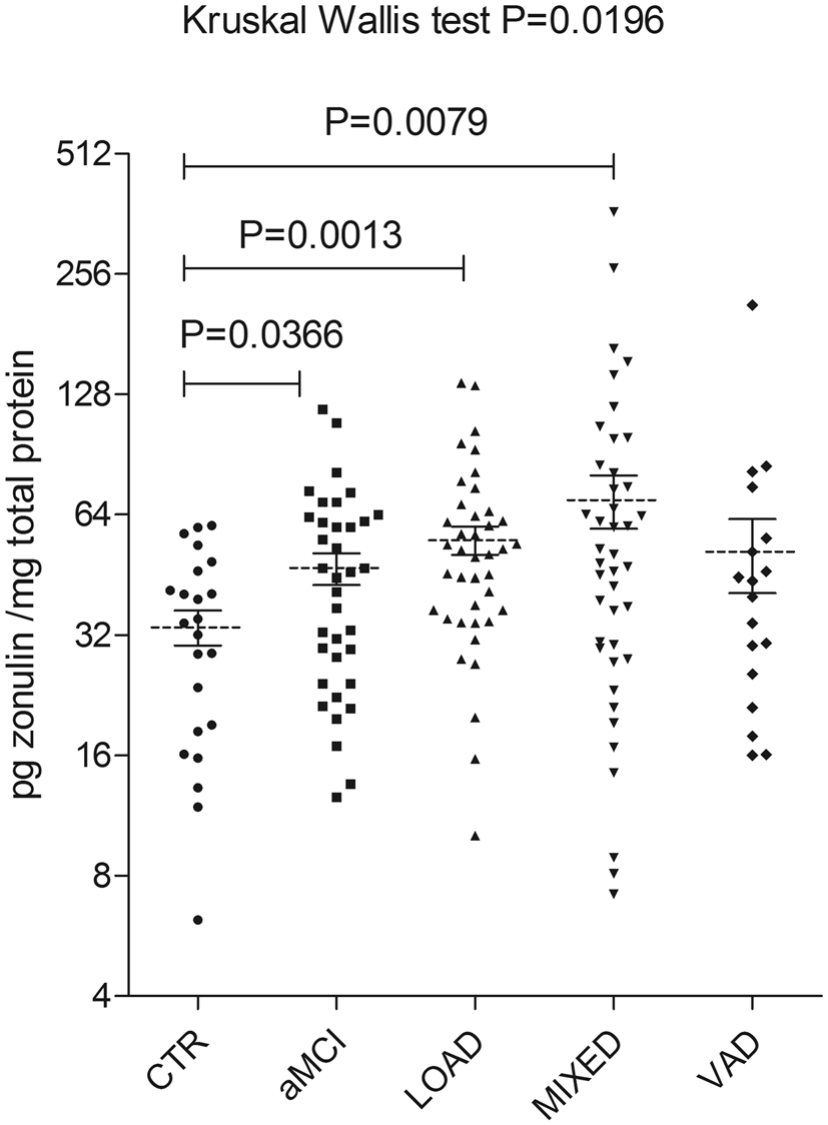
Serum zonulin levels in patients with different types of dementia. Zonulin concentration for each patient was measured using Cusabio ELISA assay and expressed as pg zonulin/mg total proteins. Zonulin values for each group of patients were reported as mean ± SE. Zonulin levels were determined in a control group (CTR; 33.5 ± 3.4); in patients with amnestic mild cognitive impairment (aMCI; 47.0 ± 4.2); in patients with mild/moderate late-onset Alzheimer’s disease (LOAD; 55.2 ± 4.4); in patients with significant vascular disease and/or focal neurological signs of memory impairment and brain atrophy (MIXED; 69.6 ± 10.6); and in a group of patients with vascular dementia (VAD; 51.6 ± 10.8). Differences in zonulin levels among study cohorts were evaluated using the Kruskal–Wallis test (*p* = 0.0169) coupled with Dunn’s Multiple Comparison as a post-test. The difference between aMCI, LOAD or MIXED vs. CTR was *p* = 0.0366 *p* = 0.0013 and *p* = 0.0079, respectively

Compared to CTR (33.5±3.4 pg zonulin/mg total proteins ± SE), the mean value of serum zonulin was 1.4 times higher in aMCI (47.0±4.2 pg zonulin/mg total proteins ± SE; *p*=0.04) patients; 1.6 times higher in LOAD (55.2±4.4 pg zonulin/mg total proteins ± SE; *p*=0.001); 2 times more abundant in MIXED dementia (69.6±10.6 pg zonulin/mg total proteins ± SE; *p*=0.008 (Fig. 2). A non-significant trend toward an increase serum in zonulin levels in VAD was also observed (51.6±10.8 pg zonulin/mg total proteins ± SE; (*p*=0.20).

### Correlations between zonulin serum levels and demographic and clinical variables

In the whole cohort (n=159 subjects) serum zonulin inversely correlated with MMSE score, years of formal education and free thyroxin (FT4), and positively correlated with hemoglobin and fasting glucose levels (Table 2). ANCOVA revealed that the difference in zonulin between CTR and LOAD, MIXED dementia or aMCI remained statistically significant even after adjustment for age, sex, education, hemoglobin, FT4, and fasting glucose (ANCOVA *p*=0.01; CTR vs. LOAD, *p*=0.01; CTR vs. MIXED, *p*=0.003; aMCI vs. controls, *p*=0.04).

**Table 2:** Correlation coefficients between zonulin/TP and age, MMSE, and clinical chemistry parameters

Variable	r	*p*
Age^#^	− 0.03	0.66
MMSE*	− 0.22	0.003
Education*	− 0.16	0.03
Hemoglobin^#^	0.17	0.03
Creatinine^#^	0.10	0.25
Total cholesterol^#^	− 0.05	0.51
hs-CRP*	0.15	0.16
Fasting glucose^#^	0.18	0.01
FT4^#^	− 0.16	0.04
TSH^#^	− 0.06	0.39

*MMSE* standardized mini-mental state examination, *TSH* thyroid stimulating hormone, *FT4* free thyroxine, *hs-CRP* high-sensitivity C-reactive protein, *TP* total proteins

*Spearman’s correlation coefficient

^#^Pearson’s correlation coefficient

### Longitudinal data: serum zonulin levels as predictors of conversion from aMCI to dementia

**Fig. 3:**
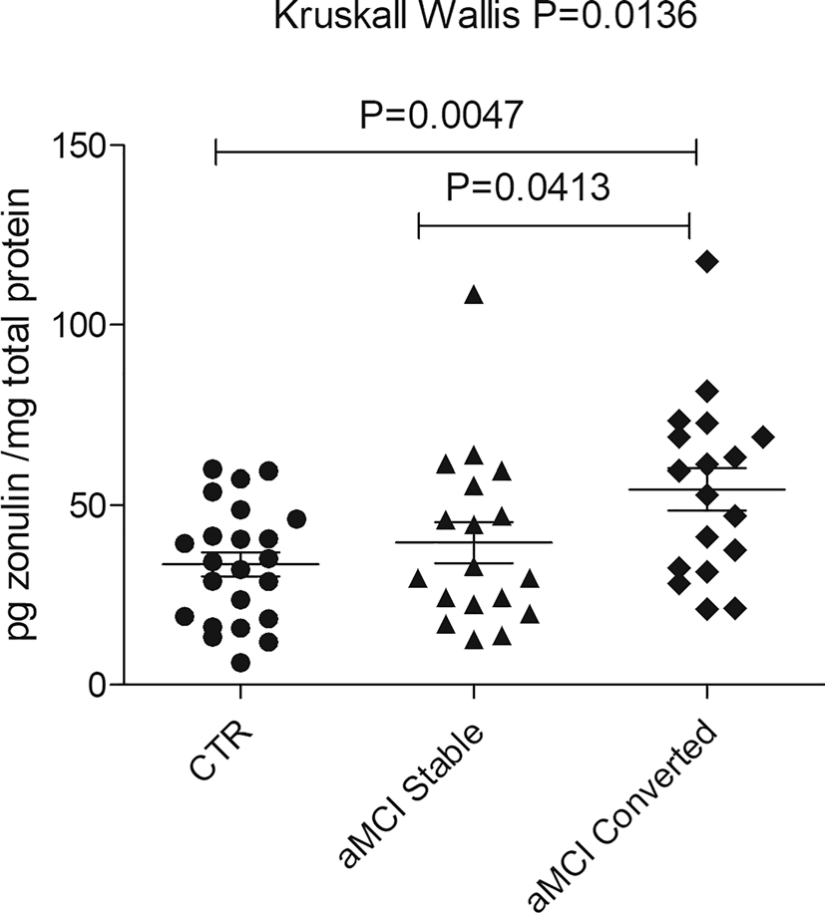
Serum zonulin levels as predictors of progression from aMCI to dementia. Zonulin concentration for each patient was measured using Cusabio ELISA assay and expressed as pg zonulin/mg total proteins. Zonulin values were reported as mean ± SE. Zonulin levels were determined in a control group (CTR; 33.5 ± 3.4 pg zonulin/mg total proteins ± SE), and two subsets of aMCI patients. The first group represents aMCI (39.6 ± 5.7 pg zonulin/mg total proteins ± SE); the second includes patients that evolved to AD, i.e., progressing from an original aMCI (54.4 ± 5.8 pg zonulin/mg total proteins ± SE). Differences in zonulin levels among groups were evaluated using the Kruskal–Wallis test (*p* = 0.0136) coupled with Dunn’s Multiple Comparison as a post-test. The difference between CTR and progressed aMCI was *p* = 0.0047; the difference between stable vs. progressed aMCI was *p* = 0.0413

We tested whether serum zonulin might be associated with the progression from aMCI to AD (Fig. 3). We subdivided aMCI patients into two subsets, one with a stable condition (n=18; age 77.7±4.5 years) and the other evolving to AD (n=18; age 78.5±2.9 years). Serum zonulin levels were significantly lower in stable aMCI (39.6±5.7 pg zonulin/mg total proteins ± SE) compared to those in patients progressing to AD (54.4±5.8 pg zonulin/mg total proteins ± SE) (*p*=0.04). Notably, this difference remained significant even after adjusting for age, sex, education, hemoglobin, FT4 and fasting glucose (ANCOVA *p*=0.01; CTR vs. aMCI converted, *p*=0.005; aMCI converted vs. stable aMCI, *p*=0.04).

## Discussion

The first finding of this study is a significant increase in serum zonulin levels in patients affected by aMCI, LOAD, or MIXED (AD + VAD) dementia, compared to healthy controls. To date, only Wang et al. have reported an increase in serum zonulin in subjects with MCI and AD [17]. The conceptual basis of our study is founded on the emerging hypothesis that a leaky gut may alter the BBB via the gut-brain axis thus playing a critical role in the development of age-related disorders [31]. Since zonulin physiologically increases also in healthy older individuals [24] we expect that a certain permeability threshold must be exceeded before we can observe any effect on cognitive functions. To understand the possible role of altered intestinal barrier, it is essential to define the timeline of events leading to dementia. So far, it is not yet clarified whether gut dysbiosis or an altered barrier is the cause or consequence of CNS disorders. In an attempt to better understand whether altered structural integrity of the intestinal barrier may precede the clinical manifestations of AD, we investigated a group of patients with aMCI. In aMCI patients, memory loss is the predominant aspect, and it is indeed associated with a high risk of evolution to AD [32]. Our finding of increased serum zonulin levels in aMCI strongly suggests that changes in gut permeability would occur before AD onset. This concept is further reinforced by the finding that serum zonulin levels are even higher in aMCI progressing to AD compared to stable aMCI. This is a new important finding, since MCI is a heterogeneous condition characterized by different possible outcomes including reversion to normal condition, stability, or conversion to overt dementia. This result suggests that zonulin, and likely other markers of gut permeability, may help to predict the clinical progression of the disease. Our results also indicate that a possible relationship between the zonulin amount and the systemic effects on permeability. Obviously, this hypothesis needs to be confirmed by in vitro studies but it provides the basis for further investigation of the dose/effect aspects of this molecule and its prognostic value in cognitive impairment.

A second and original finding is that serum zonulin levels were not significantly increased in VAD. Once confirmed, this new data would support the concept that a dysregulation of intestinal-epithelial barrier and/or BBB would be a specific event of AD-related neurodegeneration. Several lines of evidence indicate that the gastrointestinal tract and the CNS are bidirectionally connected through the microbiota-gut-brain axis [31]. Our results suggest that impaired permeability may be associated with a full (LOAD) or partial (MIXED) neurological AD phenotype, rather than with dementia secondary to cerebrovascular disease (i.e. cerebral small/large vessel disease).

Overall, our findings suggest that an increase in serum zonulin indicates the occurrence of pathological processes that are active since the preclinical stage of AD. In this line, the concept linking altered intestinal and blood-brain barriers to neurodegeneration has been postulated during the 60s in early studies showing the occurrence of neuronal loss in patients with celiac disease, a paradigmatic condition characterized by increased gut permeability [33]. Kinney et al. performed an accurate post-mortem evaluation of brain specimens from celiac disease patients and demonstrated neurodegenerative features in the cerebellum, deep grey matter, brain stem, and spinal cord [34]. This and other evidence inspired the idea of a bidirectional relationship between the brain and gut microbiota currently referred to as the “gut microbiota-brain axis” [35]. Although the pathogenetic interplay between a leaky gut and neurodegeneration is still far from being elucidated, our data provide a basis for studies aimed at exploring the occurrence and role of barrier dysfunction in patients with AD and other forms of dementia. The increased serum zonulin in aMCI, LOAD and MIXED (AD+VAD) dementia may occur because of altered intestinal homeostasis in AD. The complex interplay between gut and brain is an emerging frontier implying that some neurological diseases can originate from gut dysbiosis via altered epithelial and brain barriers. A dysbiotic, pro-inflammatory, gut microbiota has been reported in various chronic systemic inflammation, and microbiota-related products can cross the BBB thereby increasing inflammation, which results in tau protein phosphorylation and increased Aβ peptides accumulation in the brain [36].

Since zonulin modulates TJs in the intestinal epithelial and vascular barriers, as well as in the BBB, increased serum zonulin levels may reflect the damage to BBB similarly to the “leaky gut”. Indeed, BBB has been involved in AD pathogenesis because its dysfunction induces a failure of Aβ transport from CNS to the peripheral circulation [37]. This represents an important feature, since the Aβ peptide accumulation in the brain of LOAD patients suggests an impairment of the clearance rather than an increased production of these aberrant peptides. Aβ accumulation occurs in familial early-onset AD patients which represents one of the main neuropathological hallmarks of this disease [38]. Moreover, BBB dysfunction can trigger neuroinflammation and oxidative stress, which enhance the activity of β- and γ-secretase promoting Aβ generation [37]. Interestingly, Minagar et al. reported a significant increase in serum zonulin (1.5 to 3 times) in patients with multiple sclerosis [39].

Finally, in line with Wang et al. [17] we found that serum zonulin negatively correlated with the MMSE score and, most important, this correlation was independent of potential confounders. MMSE is the most commonly used test to assess memory and other mental abilities, and a low score identifies patients with worse cognitive function. An independent correlation between serum zonulin and MMSE score further supports a relationship between zonulin and cognitive deterioration in elderly people.

Some weaknesses of this study should be acknowledged. First, we cannot exclude that some biases or unmeasured confounders might limit the reliability of our results. Second, a single time point of zonulin assessment precluded the evaluation of its trend over time in subjects evolving or not to dementia; the lack of cognitive and functional trajectory data limits an exhaustive interpretation of our results. Third, in our study LOAD diagnosis was established by NIA-AA criteria with intermediate probability [20] based on clinical, structural (MRI) and FDG-PET features, although cerebrospinal fluid markers were not available. Finally, the results should be confirmed by large-cohort studies due to the limited number of patients with aMCI and VAD diagnosis enrolled in this research. The strength of this study was the longitudinal design and the highly standardized clinical follow-up. To our knowledge, this is the first longitudinal attempt to evaluate serum zonulin as a possible predictive biomarker of dementia-related diseases.

## Conclusions

We found that serum zonulin levels were significantly increased in patients with aMCI, LOAD and MIXED dementia, but not in patients with VAD. Moreover, zonulin levels were significantly higher in aMCI progressing to AD when compared with stable ones. Overall, these findings suggest that a dysregulation of intestinal-epithelial barrier and/or BBB may be an early specific event in AD-related neurodegeneration.

## Data Availability

Raw data and derived data supporting the findings of this study are available from the corresponding author on reasonable request.
